# Belladonna in Whooping-Cough

**Published:** 1869-08

**Authors:** B. S. Woodworth

**Affiliations:** Fort Wayne


### BELLADONNA IN WHOOPING-COUGH.

By DR. B. S. WOODWORTH, Fort Wayne.

I heard Brown-Sêquard say, in a speech or lecture before the American Medical Association, at Baltimore, a few years ago, that whooping-cough could be cured in three days by belladonna. But that in order to do this it would be necessary that the physician should stay in the house of the patient and watch the effects of the medicine. I took notes of this lecture at the time, and on the first opportunity I prescribed belladonna in doses that I had not previously dared to do. I became convinced, however, that this drug was signally efficacious in whooping-cough, and I have never failed since that time to give the medicine, not only in whooping-cough, but in many cases of cough among adults that seemed to depend principally upon nervous derangements. A recent epidemic of whooping-cough in this city has given me an opportunity to witness the effects of belladonna in a larger number of cases than I have previously witnessed it; and I have no more doubt of the specific influence of it than I have of that of quinine in intermittent, or of ergot in producing contractions of the uterine muscles. By the way, although some have denied that the specific effects of the latter are certainly to be relied upon, I must truly say that I *never*, after a practice of thirty years, knew it to fail.

In the present epidemic I have treated about fifty cases—all but one were uncomplicated with other diseases. The exceptional case, a child four years old, had capillary bronchitis, and possibly very circumscribed pneumonia, and for several days death seemed imminent, but recovery took place. Most of the cases had had the usual symptoms of bronchitis for several days before I prescribed for them—in short, the disease was fully developed. I began by prescribing the extract in as large doses as I thought the patient would bear, and increasing it at every successive dose until the pupils were fully dilated, and then kept them dilated, being careful to tell the friends to watch the effect and omit the medicine in case any dangerous symptom supervened. I have never seen any ill effects from it. In a majority of the cases that characteristic scarlet flush or efflorescence appeared, and with it an abatement of the cough, or of its spasmodic character. In a few cases I gave opium with the belladonna, or alternately. In that case the dilation of the pupils will not be witnessed, if they be given in about the medium dose of each—they balancing (not neutralizing) each other. I believe it is now generally conceded that those narcotics, which we call mydriatics, are antidotes (or *nearly* so) to those that produce contraction of the pupil, and *vice versa.* But, perhaps, more experiments, or more experience, are wanting to verifiy this. I, however, think it probable that we may find it advantageous to prescribe the two together sometimes, thus avoiding the bad effects of either, while the good are obtained. This is no new principle in medicine, I am aware, and for a long time I have acted on that principle in reference to quinine and opium—considering one an anti-congestive, while the other is congestive in its effect.

Now, although I have not proved Brown-Sêquard's saying, that whooping-cough can be cured in three days, I verily believe it can be cut short; and there is no more need of whooping-cough continuing for months than there is for ague continuing an indefinite length of time when plenty of quinine can be found. —*Western Journal of Medicine.*

